# Trends in cancer burden in Pakistan: a 30-year analysis from the Global Burden of Disease Study (1990–2019)

**DOI:** 10.3332/ecancer.2026.2060

**Published:** 2026-01-16

**Authors:** Kamlesh M Bhojwani, Ahmed Raheem, Urooba Tariq Khan, Fahad Javid, Daniyal Tanweer, Nawal Rehmani, Nadeem Ullah Khan, Saqib Raza Khan

**Affiliations:** 1 Department of Emergency Medicine, Aga Khan University Hospital, Karachi 74800, Pakistan; 2 Department of Medicine, Aga Khan University Hospital, Karachi 74800, Pakistan; 3 Department of Undergraduate Medical Education, Aga Khan University, Karachi 74800, Pakistan; 4 Division of Medical Oncology, Department of Oncology, Schulich School of Medicine & Dentistry, Western University, London, ON N6A 3K7, Canada; 5 Verspeeten Family Cancer Centre, London Health Sciences Centre, London, ON N6A 5W9, Canada

**Keywords:** cancer, global burden of disease, cancer incidence, cancer mortality, cancer DALYs

## Abstract

**Background:**

Cancer is a leading cause of mortality worldwide, with significant regional disparities in its burden. In Pakistan, the sixth most populous country, cancer contributes significantly to the healthcare burden, yet comprehensive epidemiological studies are limited.

**Methods:**

A population-based analysis was conducted using Global Burden of Disease (GBD) 2019 data. Cancer metrics, including incidence, mortality and disability-adjusted life years (DALYs), were extracted and stratified by age, gender, cancer type and geographic region. Annual average percent changes (AAPCs) were calculated to evaluate temporal trends. This study aimed to analyse trends in cancer incidence, mortality and DALYs in Pakistan from 1990 to 2019, using data from the GBD study.

**Results:**

Total cancer incidence increased from 1.28 million cases (1990) to 2.77 million cases (2019), with an annual average percent change (AAPC) of 0.11%. Mortality rose from 73,424 deaths (age-standardised rate (ASR): 122.16) to 179,773 deaths (ASR: 153.52) (AAPC: 0.79%) and DALYs increased from 2.41 million (ASR: 3,363.75) to 6.27 million (ASR: 3,439.9) (AAPC: 0.85%). Breast cancer remained the leading contributor to the disease burden, accounting for 51,438 cases, 32,118 deaths and 1.12 million DALYs in 2019. Pancreatic cancer showed the highest growth in incidence (AAPC: 3.39%), while ovarian cancer exhibited the largest increases in mortality (AAPC: 3.16%) and DALYs (AAPC: 5.85%). Punjab reported the highest burden, with 1.49 million cases in 2019. Female cancer incidence and mortality consistently exceeded male figures, with an AAPC of 0.15% in incidence and 1.07% in mortality. Age-specific analyses revealed that leukaemia was the leading cancer in children aged 0–14, while lung cancer dominated among males over 50 years.

**Conclusion:**

The rising cancer burden in Pakistan underscores the need for a robust, nationwide cancer registry and targeted interventions. Public health policies must address risk factors such as smoking, hepatitis infections and socio-economic disparities to curb this escalating crisis.

## Introduction

Cancer is one of the leading causes of death and morbidity globally. According to estimates by World Health Organisation (WHO), cancer is the leading cause of death in 112 of 183 countries before the age of 70 [1, 2]. The International Agency for Research on Cancer (IARC) GLOBOCAN 2022 update estimates approximately 20 million new cases and 9.7 million cancer-related deaths worldwide in 2022. The lifetime risk of developing cancer is roughly one in five, with mortality risks of one in nine for men and one in 12 for women. Lung cancer leads globally as the most diagnosed cancer (2.5 million cases, 12.4% of total) and the primary cause of cancer mortality (18.7%), followed by breast, colorectal, prostate and stomach cancers for incidence and colorectal, liver, breast and stomach cancers for mortality. Among women, breast cancer is the most common, while lung cancer dominates in men for both incidence and mortality [3, 4].

With demographic shifts, annual new cases are projected to soar to 35 million by 2050, emphasising the urgent need for prevention strategies targeting tobacco use, obesity and infections [3, 4]. In South Asia, including Pakistan, cancer patterns diverge from global trends. GLOBOCAN-based analyses reveal lower crude incidence and mortality rates compared to global averages, but a higher mortality-to-incidence ratio (MIR ~0.65 versus ~0.52 globally), signaling poorer survival outcomes. Breast and cervical cancers predominate in women, while lung, colorectal and head-and-neck cancers are prevalent in men, driven by tobacco use (both smoked and smokeless) and infections like hepatitis B/C and Human Papillomavirus (HPV). Pakistan mirrors these regional trends, with breast cancer leading overall, followed by significant burdens from lung and head-and-neck cancers. Late-stage diagnoses and limited healthcare infrastructure exacerbate high MIRs [3, 5].

Pakistan, a resource-constrained country with the world’s sixth-largest population, faces a dual burden of non-communicable diseases (e.g., hypertension, diabetes and ischemic heart disease) and infectious diseases, straining its healthcare system [6, 7]. A 2019 analysis of Pakistan’s cancer burden, drawn from national registry data, highlights its substantial and varied impact across sex, age and geography. Breast cancer tops the list (24.1% of cases), followed by oral cavity (9.6%), colorectum (4.9%), esophagus (4.2%) and liver (3.9%). Sex-specific patterns show oral cavity (14.9%), colorectum (6.8%), liver (6.4%), prostate (6.0%) and lung (6.0%) as the leading cancers in males, while breast cancer overwhelmingly dominates in females (41.6%), followed by oral cavity (6.9%), cervix (5.5%) and uterus (4.7%). Age distribution skews toward middle-aged adults (~43%), followed by older (~30%) and younger adults (~20%). Among children and adolescents, central nervous system tumours, leukaemia (18.7%) and Hodgkin lymphoma (17.3%) are most common. Notably, about 30% of cases are diagnosed at advanced stages (III–IV), reflecting delays in presentation. Geographically, Punjab (~40.4%) and Sindh (~32.2%) account for most registered cases, likely due to concentrated diagnostic and registry services [8].

Risk factors in Pakistan align with regional patterns. Tobacco, including smokeless forms like areca/betel quid, is a major driver of oral cavity cancer, while smoking fuels lung cancer. Infections, such as hepatitis B/C (linked to liver cancer) and HPV (linked to cervical cancer), play a significant role. Lifestyle and metabolic factors, including rising obesity and diabetes, further contribute, alongside environmental risks like ambient air pollution. Addressing these risk factors, alongside improving early detection and healthcare access, is critical to mitigating the growing cancer burden in Pakistan and globally [6, 8, 9].

Considering the rapid increase of cases and the associated morbidity and mortality, Pakistan set up its National Action Plan for non-communicable diseases and partnered with the International Atomic Energy Agency (IAEA), WHO and the IARC. The National Cancer Registry of Pakistan, which came as an eventual result of the collaboration of IAEA and WHO, comprises of 'Punjab Cancer Registry, 'Karachi Cancer Registry', 'Pakistan Atomic Energy Commission Cancer Registry', Armed Forces Institute of Pathology Cancer Registry, Nishtar Medical University Hospital Multan and Shifa International Hospital, Islamabad. Analysis found 45.13% of cases of cancer were from Punjab, 26.83% from Sindh, 16.46% from Khyber Pakhtunkhwa and 3.52% from Baluchistan [10].

The global burden of disease (GBD) study is a comprehensive effort at understanding and reporting the changes in disease burden across countries. Started as a single World Bank-commissioned study in 1990, the most recent GBD 2019 study has been published in October-2020. The data collected is accessible for free and has allowed for global, national and regional burden estimate studies for multiple diseases [10, 11]. The GBD also reports on 87 risk factors associated with multiple diseases. The GBD study provides a large platform for estimation studies. Pakistan lacks a concerted national cancer care registry that is truly representative and updated with the disease burden. The 2019 update of GBD offers estimates at the subnational level for Pakistan, which has helped with estimating the progress Pakistan has made in reducing disease burden [12].

Our aim for this paper is to do a comprehensive analysis of cancer burden in Pakistan. We also aim to stratify cancer types according to incidence, deaths and disability-adjusted life years (DALYS), geography/region, age groups and gender. With the estimated high burden of disease, it is imperative to understand the landscape of the disease. We hope to inform policy makers, local authorities and researchers for a more targeted and effective approach to cancer at a national level.

## Methodology

### Study design

This is a population-based study focused on the cancer burden in Pakistan, utilising data from the GBD database, developed by the Institute for Health Metrics and Evaluation (IHME).

### Data source and setting

We extracted data on cancer incidence, mortality, DALYs, prevalence rate and death rate for Pakistan from the GBD study for the period 1990–2019. The data were stratified by age group, gender and cancer subtype. While reporting metrics for different cancer subtypes, the subtype ‘Other neoplasms’ was excluded from the analysis.

### Data extraction

Cancer data for Pakistan from 1990 to 2019 was extracted using the Global Health Data Exchange (GHDx) query tool. The dataset was further divided by gender, age group and province. Specific selections from the GBD database included the following categories: for ‘GBD Estimate,’ Cause of death or Injury was chosen; for ‘Measure,’ Incidence, Prevalence, Deaths and DALYs were selected; for ‘Metric,’ both Number and Rate were included; for ‘Cause,’ Neoplasms (all except 'Other Neoplasms') were selected; for ‘Location,’ Pakistan was chosen; for ‘Age,’ the groups included All ages, less than 28 days, 28–364 days, 1–4 years, 5–9 years, 10–19 years, 20–54 years and 55+ years; for ‘Sex,’ both male and female were included and for ‘Year,’ the all years ranging from 1990 to 2019 were selected.

As this study used publicly available secondary data, ethical approval was not required. The complete data set used for the analysis in this research is publicly available from the GBD 2019 study by the IHME, and it can be extracted from the GHDx query tool [13].

### Statistical analysis

Data analysis was performed using Microsoft Excel 2013, R-Studio (version 4.2.2) and Business Intelligence software. Poisson regression analysis was applied to evaluate trends across cancer types. Percentage changes in incidence, prevalence and deaths were calculated for 2019 compared to 1990. The top three cancers with the highest burden were analysed by age group based on absolute numbers and rates per 100,000 population. DALY estimates were obtained directly from the GBD 2019 database using the GHDx query tool, which calculates DALYs as the sum of years of life lost and years lived with disability for each cancer type, stratified by age, sex and location, based on standardised GBD methodology. Age-standardised DALY rates and their 95% uncertainty intervals (UIs) were extracted for Pakistan for each year from 1990 to 2019, and temporal trends were analysed using Poisson regression to compute annual average percent changes (AAPCs) with 95% confidence intervals (CIs).

## Results

Table 1 shows the incidence cases and age-standardised rates (ASRs) per 100,000 for various cancer types in Pakistan from 1990 to 2019. The total cancer cases in Pakistan increased from 1,283,332 cases with an ASR of 1566.48 in 1990 to 2,769,507 cases with an ASR of 1,617.33 in 2019, exhibiting an AAPC of 0.11% between 1990 and 2019. Pancreatic cancer exhibited the highest annual average percent change (AAPC) of 3.39% (95% CI: 3.36–3.43) between 1990 and 2019, indicating the greatest increase. Conversely, chronic myeloid leukaemia showed the greatest decline with an AAPC of −0.77% (95% CI: −0.81 to −0.73) during the same period. In 2019, breast cancer had the highest incidence with 51,438 cases (95% UI: 37,937–69,293) and an ASR of 38.35 (95% UI: 28.78–50.97), increasing by 2.26% (95% CI: 2.22–2.30) annually since 1990. Lip and oral cavity cancer also exhibited a high burden with 28,579 cases (95% UI: 22,907–35,935) and an ASR of 21.93 (95% UI: 17.83–27.56) in 2019, rising by 1.00% (95% CI: 0.97–1.04) annually. Mesothelioma had the lowest incidence in 2019 with 237 cases (95% UI: 146–379) and an ASR of 0.20 (95% UI: 0.13–0.31), but increased by 1.07% (95% CI: 1.04–1.09) annually since 2010.

Among the provinces, Punjab had the highest incidence in 2019 with 1,489,130 cases and an ASR of 1,630.1, increasing by 0.11% (95% CI: 0.1–0.11) annually since 1990. Islamabad exhibited the highest AAPC of 0.23% (95% CI: 0.22–0.23) between 1990 and 2019. Regarding gender, the incidence was higher among females, with 1,479,890 cases and an ASR of 1,694.51 in 2019, increasing by 0.15% (95% CI: 0.15–0.16) annually since 1990.

The total cancer deaths in Pakistan increased from 73,424 with an ASR of 122.16 in 1990 to 179,773 with an ASR of 153.52 in 2019, exhibiting an AAPC of 0.79% (95% CI: 0.78–0.81) between 1990 and 2019 (Table 2). Ovarian cancer exhibited the highest AAPC of 3.16% (95% CI: 3.12–3.18) during this period, with deaths increasing from 4,148 in 1990 to 7,002 in 2019. Conversely, other leukaemia and chronic myeloid leukaemia showed the greatest decline with an AAPC of −0.99% (95% CI: −1.01 to −0.96) and −0.77% (95% CI: −0.79 to −0.74), respectively. In 2019, breast cancer had the highest death toll with 32,118 cases and an ASR of 26.34, increasing by 1.71% annually since 1990. Lip and oral cavity cancer also contributed significantly, with 17,567 deaths and an ASR of 14.72 in 2019, rising by 0.68% (95% CI: 0.65–0.7) annually during the same period.

Among the provinces, Punjab had the highest death toll in 2019 with 107,232 cases and an ASR of 162.94, increasing by 0.73% (95% CI: 0.71–0.75) annually since 1990. Khyber Pakhtunkhwa and Balochistan exhibited the highest AAPC of 1.01% between 1990 and 2019. Regarding gender, the death toll was higher among females, with 91,533 cases and an ASR of 158.04 in 2019, increasing by 1.07% (95% CI: 1.05–1.09) annually since 1990.

The total cancer-related DALYs in Pakistan increased from 2,414,828 cases with an ASR of 3,363.75 in 1990 to 6,268,083 cases with an ASR of 3,439.9 in 2019, exhibiting an AAPC of 0.85% (95% CI: 0.82–0.88) between 1990 and 2019 (Table 3). Ovarian cancer exhibited the highest annual average percent change (AAPC) of 5.85% (95% CI: 5.81–5.88) between 1990 and 2019, indicating the greatest increase. Conversely, other leukaemia showed the lowest increase with an AAPC of 0.48% (95% CI: 0.38–0.57) during the same period. In 2019, breast cancer had the highest burden with 1,119,378 DALYs (95% UI: 847,412–1,520,925) and an ASR of 783.3 (95% UI: 597.3–1,054.53). Lip and oral cavity cancer also exhibited a high burden with 594,089 DALYs (95% UI: 471,317–754,897) and an ASR of 421.87 (95% UI: 338.19–535.02) in 2019. Non-melanoma skin cancer (basal-cell carcinoma) had the lowest burden with 1 DALY (95% UI: 0–1) and an ASR of 0 in 2019.

Among the provinces, Punjab had the highest DALYs in 2019 with 3,717,719 cases and an ASR of 3,538.2, increasing by 0.75% (95% CI: 0.73–0.77) annually since 1990. Baluchistan exhibited the highest AAPC of 1.12% (95% CI: 1.08–1.17) between 1990 and 2019. Regarding gender, the burden was higher among females, with 3,223,297 DALYs and an ASR of 4,012.8 in 2019, increasing by 1.07% (95% CI: 1.04–1.09) annually since 1990, while males showed a lower AAPC of 0.67% (95% CI: 0.64–0.69).

Table 4 shows the most common cancers in various regions of Pakistan based on prevalence and death rate, as of 2019. Breast cancer is the most prevalent cancer type in all regions of Pakistan. Despite its high prevalence, it ranks 5th in terms of death rate in most regions except in Balochistan and Islamabad Capital Territory where it is 6th and 7^th^, respectively. Lip and oral cavity cancer ranked second across Pakistan. Kidney cancer and multiple myeloma, despite a lower prevalence, show a notably high death rate. Some cancers, like larynx cancer and prostate cancer, show the opposite trend with high prevalence rates but relatively lower death rates.

**Figure 1.**
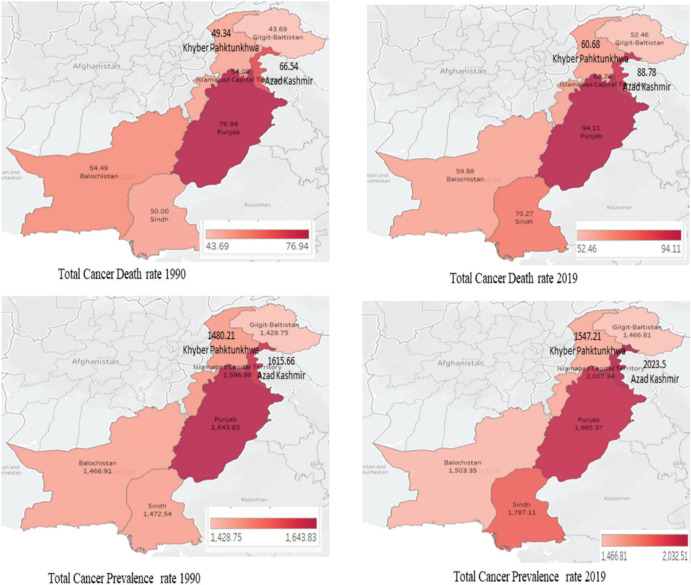
Cancer death and incidence rates in Pakistan's states: 1990 versus 2019 comparison.

Figure 1 shows a map comparing cancer death and prevalence rates in different cities across Pakistan between 1990 and 2019. Punjab and Sindh emerged as hotspots with high cancer rates. The highest cancer death rates in 2019 were seen in the provinces of Punjab and Azad Kashmir, with rates of around 94 and 89, respectively, per 100,000 population. These same provinces also had the highest cancer prevalence rates in 2019, with around 2,000 cases, respectively, per 100,000 people. Between 1990 and 2019, Sindh saw the biggest increase in both death rate (from around 50 to over 70 per 100,000) and prevalence rate (from around 1,472 to over 1,787 per 100,000). The province with the lowest cancer death and prevalence rates was the northern region of Gilgit–Baltistan. Their rates remained relatively stable from 1990 to 2019. In summary, the data show substantial geographic variation in cancer burden across different cities in Pakistan.

Table 5 describes the top three most common cancers in terms of number of incidence, deaths and DALYs among different age groups in Pakistan. Among all age groups, breast cancer emerged as the leading cause of cancer incidence, deaths and DALYs in Pakistan. For ages 20–54 years and 55 years and above, the top three cancers contributing to incidence were breast cancer, lip and oral cavity cancer and tracheal, bronchus and lung cancer. This pattern was mirrored in the rankings for deaths and DALYs across these age groups. However, the rankings varied significantly for younger age groups. For instance, among children aged less than 28 days, brain and central nervous system cancer, other leukaemia and liver cancer were the top three contributors to incidence, deaths and DALYs. In the age group of 28–364 days and 1–4 years, other leukaemia, brain and central nervous system cancer, and acute lymphoid leukaemia dominated the rankings. For ages 5–9 years, acute lymphoid leukaemia, brain and central nervous system cancer and Hodgkin lymphoma were the leading cancers, while Hodgkin lymphoma, brain and central nervous system cancer and non-Hodgkin lymphoma ranked highest among those aged 10–19 years.

**Figure 2.**
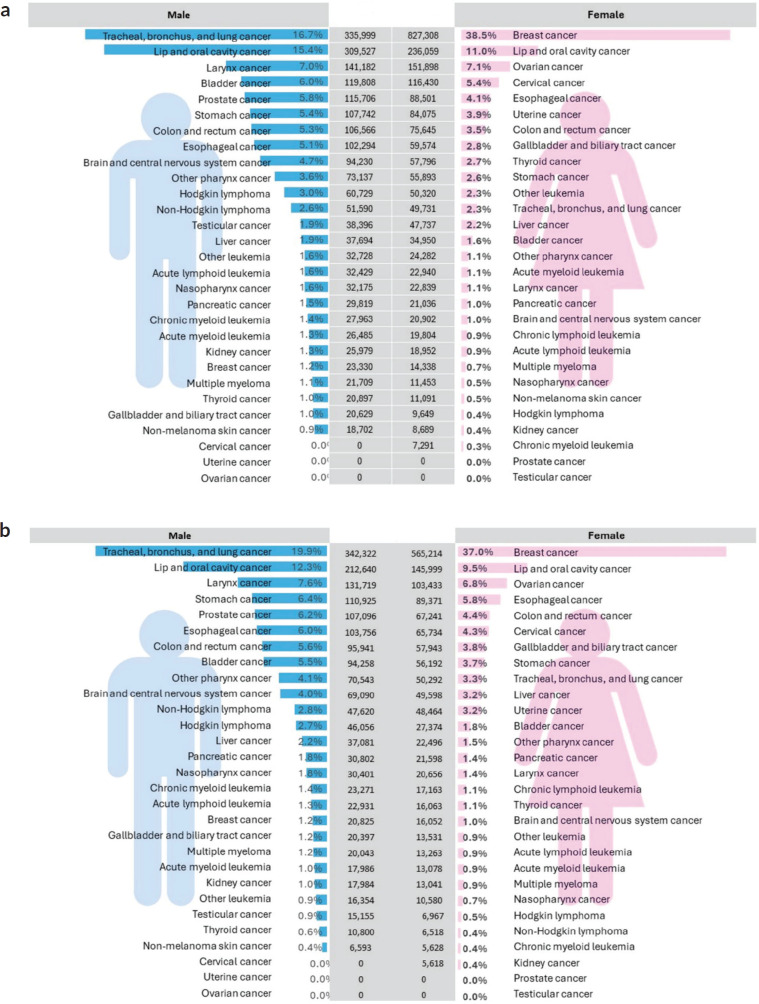
(a): Incidence counts for major cancer types in males and females. (b): Death count for major cancer types in males and females.

Figure 2 highlights significant gender disparities in the burden of various cancers. Among males, tracheal, bronchus and lung cancer emerge as the leading cause of both new cases and deaths, followed by lip and oral cavity cancer and larynx cancer. In contrast, breast cancer stands out as the predominant cancer afflicting females, in terms of incidence as well as mortality. Lip and oral cavity cancer and ovarian cancer rank second and third, respectively, in terms of incidence as well as mortality.

## Discussion

Cancer remains a major public health challenge in Pakistan, with a rising burden in terms of incidence, mortality and DALYs. The total cancer incidence more than doubled from 1.28 million cases in 1990 to 2.77 million cases in 2019, with an annual average percent change (AAPC) of 0.11%. Mortality due to cancer increased from 73,424 deaths in 1990 to 179,773 in 2019 (AAPC: 0.79%), while DALYs rose from 2.41 million to 6.27 million (AAPC: 0.85%) during the same period.

The cancer burden in Pakistan should be viewed within the broader context of South Asian countries, where cancer risk is increasing and accounts for approximately 25% of all deaths [14]. Like other countries in the Asian Pacific region, Pakistan's relatively low cancer survival rates can be attributed to multiple factors, including limited awareness, inadequate healthcare infrastructure and challenging socioeconomic conditions [15]. This pattern aligns with global observations showing disproportionate cancer burden distribution, where, although higher human development index countries report greater incidence, lower-resource countries like Pakistan often face higher mortality rates [16].

Across Pakistan’s provinces, the cancer burden varies widely, revealing stark regional differences. Punjab stands out as the most affected region, reporting 1.49 million cancer cases in 2019, with Sindh and Khyber Pakhtunkhwa also showing significant burdens. These disparities likely stem from a mix of factors—larger populations in some areas, the effects of urban growth, differing environmental risks and uneven access to quality healthcare [17]. Supporting this, recent data from Lahore highlights a sharp increase in cancer cases among individuals aged 41–50, particularly breast cancer in women aged 41–60 [17]. This uneven spread highlights why we need focused efforts and smarter resource distribution.

### Specific cancer trends

Breast cancer emerged as the most prevalent cancer across various age groups in Pakistan, with 51,438 reported cases in 2019 and an ASR of 38.35 per 100,000, reflecting an annual increase of 2.26%. What is striking is that more than half of breast cancer diagnoses and nearly two-thirds of related deaths occur in less developed regions, underscoring the disproportionate burden faced by low- and middle-income countries [1]. The disease’s prevalence is closely tied to levels of human development, with metabolic risks contributing to 31.98% of attributable deaths in 1990, a figure that rose to 46.87% by 2019 [18]. Other contributing factors, such as alcohol consumption, tobacco use, poor dietary habits and sedentary lifestyles, further complicate the disease’s epidemiology [18, 19]. Sure, wealthier nations see higher incidence rates, but in developing regions, the larger populations result in a greater overall burden of disease [20]. These findings highlight the urgent need for targeted interventions that address modifiable risk factors and enhance early detection efforts, particularly in countries like Pakistan with similar socio-economic challenges.

Lip and oral cavity cancer has also become a major public health issue in Pakistan, with 28,579 cases reported in 2019, an ASR of 21.93 per 100,000 and an annual increase of 1.00%. This trend is consistent with global data, which highlights the significant burden of these cancers, especially in regions with lower socioeconomic development [21]. Worldwide, lip and oral cavity cancers account for approximately 3.2% of all cancer-related deaths, with considerable variation in incidence and mortality rates across different geographic areas [21]. Our findings support international research that identifies key risk factors, including tobacco use, alcohol consumption, HPV infections and betel quid chewing, as major drivers of the disease [21–23]. Pakistan, though, stands out with one of the highest age-standardised mortality rates for this cancer type emphasising the urgent need to act fast [21]. The ongoing high prevalence of lip and oral cavity cancers in Pakistan underscores the necessity for comprehensive strategies that address modifiable risk factors, such as tobacco control, lifestyle changes and enhanced early detection programs.

Pancreatic cancer showed the highest annual average percent change of 3.39% between 1990 and 2019 in Pakistan, a trend that aligns with global projections of increasing pancreatic cancer burden. Epidemiologic models suggest that by 2030, pancreatic cancer is expected to become the second leading cause of cancer-related mortality in the United States, emphasising the critical nature of this disease [24]. The prognosis remains exceptionally challenging, with a low 5-year survival rate of approximately 12% and less than 20% of patients eligible for potentially curative surgical resection [24]. Cigarette smoking emerges as the most well-established risk factor, with studies indicating that it could contribute to 15%–25% of pancreatic cancer cases across various populations [25]. The limited treatment options and minimal effectiveness of current therapies for metastatic pancreatic cancer underscore the urgent need for comprehensive research and prevention strategies [24]. An additional concern is the emergence of Early Onset Pancreatic Cancer, which affects approximately 10% of patients aged 50 or younger, presenting a complex clinical challenge with limited understanding of its specific risk factors [26]. These findings collectively highlight the critical importance of primary prevention, particularly tobacco control and the necessity for continued investment in basic, translational and clinical research to address this devastating disease.

Ovarian cancer has shown a concerning upward trend in Pakistan, with the highest annual average percent change of 3.16% in mortality and 5.85% in (DALYs) between 1990 and 2019. Globally, ovarian cancer is a significant gynecologic malignancy, ranking seventh in incidence among all cancers and eighth in cancer-related deaths among women [27]. In 2020, global estimates reported approximately 314,000 new cases and 207,000 deaths, highlighting its substantial impact on women’s health worldwide [1]. The disease has a global incidence rate of 6.6 per 100,000 and a mortality rate of 3.9 per 100,000, though these figures vary significantly across regions [1]. The risk factors for ovarian cancer are complex, with studies indicating that higher parity may increase risk, while factors such as oral contraceptive use and tubal ligation may offer protective benefits [28]. Although ovarian cancer does not rank as high as lung, colorectal or stomach cancers in terms of overall incidence and mortality, it remains a critical health issue, particularly due to its high fatality rate among gynecologic cancers [27]. The rising trends observed in our study are consistent with global patterns, underscoring the urgent need for improved screening programs, early detection strategies and targeted interventions to address the growing burden of ovarian cancer.

### Contributing factors

Pakistan’s cancer burden is driven by a mix of socioeconomic, environmental and behavioural factors. Tobacco use, including smoking and betel quid, is a major risk factor, linked to 15%–25% of pancreatic cancer cases and significantly contributing to oral and pharyngeal cancers [23, 25]. The WHO estimates that 40% of Pakistan’s cancer risk could be reduced through tobacco cessation [17]. Chronic infections like hepatitis B and C further elevate cancer risk, particularly for liver cancer, worsened by interactions with metabolic and environmental factors in a resource-strained setting [29]. Socioeconomic challenges, including low development levels, limited healthcare access and inadequate early detection, increase mortality, especially for lip and oral cavity cancers [21]. Poor nutrition, contaminated food and limited access to balanced diets are significant contributors to cancer development [29].

### Public health implications

The growing cancer crisis in Pakistan is not just a health issue—it is a call to action. With rising cancer rates, increasing deaths, and the toll it takes on people’s lives (measured by disability-adjusted life years or DALYs), it is clear that Pakistan’s healthcare system needs a major overhaul. But here is the thing: cancer does not affect everyone equally. Some provinces, age groups and genders are hit harder than others. To address this effectively, Pakistan must prioritise prevention, strengthen early detection efforts and enhance treatment capabilities, tailoring strategies to our unique circumstances [30]. Our findings highlight a few critical intervention areas.

### Early detection and screening programs

Timely diagnosis could prevent 40% of cancer deaths in Pakistan, yet screening remains inaccessible to 85% of the population [17]. Strategic priorities include:

- High-risk targeting: Implement mammography screening for women aged 40–60 in urban centers, coupled with mobile clinics for rural areas.
- Province-specific approaches: e.g., Sindh’s hepatitis-endemic regions require annual liver ultrasounds.
- Community engagement: Train Lady Health Workers to conduct oral visual inspections in high-*paan*-use areas like Khyber Pakhtunkhwa.

### Healthcare system strengthening

Pakistan’s cancer infrastructure suffers from urban-rural disparities and fragmented care. Key measures:

- Tiered service expansion: Establish new tertiary cancer centers (Balochistan, Gilgit-Baltistan prioritised) linked to district hospitals via tele-oncology networks so that patients, no matter where they live, can get the care they need without delay.
- Diagnostic capacity: Equip 50% of DHQ hospitals with mammography and CT scanners by 2030, using the Sehat Sahulat Program’s financing model (a social health insurance initiative by the government in Pakistan).

### Primary prevention initiatives

Many cancers in Pakistan are linked to things we can change—like smoking, poor diet, lack of exercise and chronic infections like hepatitis B and C. Prevention is always better than cure and when it comes to cancer, there is a lot we can do.

- **Tobacco control**: Enforce graphic health warnings on *gutka* packaging (following India’s precedent) and increase excise taxes to ≥70% of retail price [31].
- **Infection prevention**: Scale up hepatitis B vaccination coverage from 75% to 90% by integrating it with the Expanded Programme on Immunisation schedules in high-risk regions (e.g., interior Sindh).
- **Public awareness**: Launch culturally adapted campaigns (e.g., Urdu/Pashto radio dramas featuring survivor stories) to combat stigma and promote symptom recognition.

### Cancer awareness and education

The persistently low cancer awareness in Pakistan represents a critical barrier to early detection and treatment adherence. Cultural stigma and misconceptions about cancer further deter individuals from seeking timely medical care. A 2017 study from Karachi found that the mean patient delay was 15.7 months. It also revealed that 55.2% of women did not seek medical advice despite detecting a breast lump because of a lack of awareness about its significance. Few women (9.4%) initially sought help from spiritual healers and complementary and alternative medicine rather than medical professionals [32]. A few key interventions could transform this landscape.

- Community-Based Education Programs
  - Develop culturally-sensitive awareness materials in regional languages (Urdu, Pashto, Sindhi) featuring local influencers.
  - Train mosque imams and school teachers in basic cancer literacy through partnerships with the National Institute of Health.
- Media Partnerships
  - Collaborate with Geo TV and HUM News for prime-time awareness dramas.
  - Launch TikTok/WhatsApp campaigns targeting youth with symptom checklists.

### National cancer registry

One of the most significant challenges in developing effective cancer policies in Pakistan is the absence of a centralised and well-maintained cancer registry. Current data collection methods are fragmented, resulting in underreporting and inconsistencies. It is hard to track how many people are getting cancer, where they are located, and what types of cancer are most common. Setting up a national cancer registry would be a game changer as it would give us the real-time data, we need to make smarter decisions and measure the impact of our efforts.

Continuous research, real-time data collection and adaptable policies will be crucial to ensuring that our interventions remain effective as new trends emerge. It is a tough job, but with the right approach, we can make a real difference.

### Strengths and limitations

This study provides a detailed 30-year look at cancer trends in Pakistan, using data from the GBD study. One of its biggest strengths is its long-term perspective, which lets us see how cancer rates, deaths and the overall impact on people’s lives (measured by DALYs) have changed over time. By using standardised GBD metrics, the study gives us a clear, globally comparable picture of Pakistan’s cancer burden. It also digs into differences by age, gender, region and cancer type, offering a well-rounded view of the problem. Another strength is the use of ASRs and annual average percent changes. These tools help us go beyond simple numbers and understand the bigger picture—how cancer is affecting public health over the long term. This kind of analysis is crucial for policymakers and researchers trying to figure out where to focus resources and how to design effective interventions. The study also shines a light on regional disparities, showing that some provinces are hit harder than others, which calls for targeted action at the local level.

That said, the study is not without its limitations. While the GBD dataset is comprehensive, it comes with challenges, especially in a country like Pakistan where cancer data collection is patchy at best. Many areas do not have centralised cancer registries, so some cases might be missed or underreported. Differences in healthcare access and diagnostic capabilities across provinces could also skew the data—some cancers might be underdiagnosed in areas with fewer resources. Plus, the study leaves out the ‘Other neoplasms’ category, which means we might be missing trends in less common or harder-to-classify cancers.

Another limitation is the lack of detailed patient-level data. Without information on individual risk factors, treatment outcomes or socioeconomic influences, it is harder to get a full picture of why certain trends are happening. While the study does a great job of identifying broad patterns, its retrospective nature means we cannot draw firm conclusions about cause and effect or dig deeper into what is driving these trends. There is also the risk of selection bias—areas with better reporting systems might seem to have higher cancer rates, even if that is not the case.

Although GBD 2021 data were available at the time of publication, this study used GBD 2019 estimates to maintain a consistent 30-year analysis period (1990–2019), providing comprehensive, standardised estimates of cancer incidence, mortality and DALYs across Pakistan’s regions and demographics. Importantly, both GBD 2019 and GBD 2021 show consistent trends, such as rising absolute cancer burden, modest changes in age-standardised mortality, similar sex differences (e.g., breast and lung cancers leading incidence in women and men, respectively), site rankings and SDI gradients. We recommend that future studies incorporate GBD 2021 to capture updated estimates and additional cancer sites [33].

Despite these challenges, this study is an important step forward in understanding Pakistan’s cancer burden. It highlights the urgent need for better cancer surveillance, more widespread screening and stronger healthcare infrastructure. By improving data collection and analysis, we can make sure future research and policies are based on accurate, representative information—and that is how we will start making real progress against cancer.

## Conclusions

This comprehensive 30-year analysis highlights a concerning rise in the cancer burden in Pakistan, with increasing trends in incidence, mortality and DALYs across most cancer types, particularly among females and in the province of Punjab. The persistent dominance of breast cancer, the alarming growth in pancreatic and ovarian cancers and the regional disparities highlight the urgent need for a robust national cancer registry. Strengthening the existing cancer registries, investing in early detection and screening programs, addressing modifiable risk factors like smoking and hepatitis and enhancing healthcare infrastructure are critical to reversing this trajectory. These findings serve as a call to action for policymakers, healthcare providers and public health stakeholders to prioritise cancer prevention and care within Pakistan’s broader health agenda.

## Abbreviations

AAPC: Average Annual Percent Change, ASR: Age-Standardised Rate, CI: Confidence Interval, DALYs: Disability-Adjusted Life Years, GBD: Global Burden of Disease, GHDx: Global Health Data Exchange, HPV: Human Papillomavirus, IAEA: International Atomic Energy Agency, IARC: International Agency for Research on Cancer, IHME: Institute for Health Metrics and Evaluation, MIR: Mortality-to-Incidence Ratio, UI: Uncertainty Interval, WHO: World Health Organisation.

## Declaration of AI and AI-assisted technologies in the writing process

In the preparation of this manuscript, Claude AI was utilised for paraphrasing to reduce the word count. AI's role did not extend to ideation, analysis or interpretations presented in the manuscript. The content generated by Claude AI was thoroughly reviewed, approved and edited by the authors. The authors take full responsibility for the final content of the manuscript.

## Conflicts of interest

The authors declare that the research was conducted in the absence of any commercial or financial relationships that could be construed as potential conflicts of interest.

## Funding

The authors declare that this research received no funding.

## Author contributions

KB led the project, drafted the manuscript and assisted with data analysis. AR performed the analysis. UK, FJ, DT and NR contributed to the main manuscript text. NK critically reviewed the manuscript. SK conceptualised and supervised the study.

## Ethical approval and consent to participate

This study was conducted using publicly available data from the GBD 2019 study by the IHME. Ethical approval was not required as no human subjects were directly involved, and no identifiable personal data were collected or used.

## Availability of data and materials

The complete data set used for the analysis in this research is publicly available from the GBD 2019 Study by the IHME and it can be extracted from the Global Health Data Exchange query tool (https://vizhub.healthdata.org/gbd-results).

## Figures and Tables

**Table 1.** Pakistan’s cancer incidence trends by type, gender and province (1990–2019).

Incidence	1990		2000		2010		2019		AAPC between 1990 and 2019 (95% CI)
	Cases (95% UI)	ASR/100,000 (95% UI)	Cases (95% UI)	ASR/100,000 (95% UI)	Cases (95% UI)	ASR/100,000 (95% UI)	Cases (95% UI)	ASR/100,000 (95% UI)	
**Cancer subtype**									
Acute lymphoid leukemia	978 (565–1,943)	0.66 (0.42–1.18)	1,439 (936–2,485)	0.79 (0.55–1.25)	2,048 1,447–3,098)	0.87 (0.63–1.26)	2,448 (1,796–3,462)	0.9 (0.67–1.25)	1.11 (0.94–1.28)
Acute myeloid leukemia	877 (655–1,265)	0.88 (0.69–1.2)	1,327 (954–1,822)	1.08 (0.85–1.34)	1,983 (1,476–2,583)	1.22 (0.96–1.53)	2,567 (1,904–3,295)	1.29 (1–1.62)	1.35 (1.28–1.4)
Bladder cancer	3,263 (2,637–3,905)	6.08 (4.91–7.3)	4,292 (3,704–4,964)	7.07 (6.09–8.24)	5,808 (4,746–6,961)	7.67 (6.26–9.16)	8,153 (6,395–10,336)	8.04 (6.35–10.16)	0.96 (0.93–1)
Brain and central nervous system cancer	2,382 (1,630–3,665)	2.19 (1.65–3.02)	3,247 (2,158–4,580)	2.54 (1.86–3.33)	4,474 (3,064–5,831)	2.67 (1.94–3.38)	5,639 (3,968–7,307)	2.78 (1.99–3.54)	0.83 (0.76–0.88)
Breast cancer	12,821 (9,291–17,372)	20.26 (14.64–27.68)	21,953 (18,197–26,101)	28.4 (23.47–34.35)	33,316 (26,420–41,371)	33.23 (26.47–40.95)	51,438 (37,937–69,293)	38.35 (28.78–50.97)	2.26 (2.22–2.3)
Cervical cancer	2,196 (1,759–2,774)	3.24 (2.61–4.11)	3,499 (2,720–4,318)	4.1 (3.21–5.02)	4,386 (3,259–5,736)	3.86 (2.89–4.95)	5,699 (4,025–8,109)	3.76 (2.69–5.28)	0.53 (0.5–0.56)
Chronic lymphoid leukemia	433 (355–547)	0.77 (0.63–0.97)	645 (538–769)	0.99 (0.82–1.19)	878 (708–1,078)	1.07 (0.87–1.32)	1,239 (984–1,619)	1.13 (0.91–1.43)	1.32 (1.29–1.35)
Chronic myeloid leukemia	938 (536–1,699)	0.98 (0.72–1.34)	1,126 (725–1,785)	1.03 (0.78–1.39)	1,268 (842–1,916)	0.91 (0.67–1.24)	1,344 (985–1,890)	0.78 (0.59–1.05)	−0.77 (−0.81 to −0.73)
Colon and rectum cancer	3,111 (2,689–3,576)	5.49 (4.74–6.33)	5,110 (4,463–5,766)	7.71 (6.71–8.72)	7,084 (5,952–8,458)	8.49 (7.11–10.06)	10,140 (8,100–12,938)	9.14 (7.33–11.63)	1.76 (1.72–1.8)
Esophageal cancer	4,004 (3,376–4,680)	6.98 (5.83–8.16)	5,737 (4,854–6,541)	8.52 (7.27–9.7)	7,101 (5,899–8,423)	8.33 (6.94–9.83)	8,911 (7,068–10,991)	7.86 (6.3–9.63)	0.44 (0.42–0.47)
Gallbladder and biliary tract cancer	1,614 (1,330–2,105)	2.88 (2.36–3.79)	2,481 (2,021–2,934)	3.83 (3.08–4.54)	2,993 (2,423–3,607)	3.7 (3–4.46)	3,824 (3,067–4,759)	3.58 (2.9–4.45)	0.74 (0.71–0.76)
Hodgkin lymphoma	1,430 (1,076–1,798)	1.66 (1.28–2.15)	2,073 (1,501–2,594)	1.87 (1.41–2.38)	2,632 (1,875–3,373)	1.75 (1.3–2.26)	3,290 (2,359–4,309)	1.67 (1.22–2.22)	0.04 (0.01–0.07)
Kidney cancer	526 (438–640)	0.78 (0.64–0.93)	877 (731–1,049)	1.12 (0.97–1.31)	1,412 (1,121–1,745)	1.39 (1.12–1.7)	2,085 (1,603–2,819)	1.57 (1.2–2.12)	2.46 (2.4–2.51)
Larynx cancer	3,569 (2,963–4,241)	6.11 (5.07–7.27)	5,097 (4,355–5,937)	7.36 (6.28–8.54)	5,969 (4,797–7,457)	6.7 (5.44–8.3)	7,381 (5,644–9,715)	6.2 (4.75–8.1)	0.08 (0.05–0.11)
Leukemia	5,685 (3,282–8,690)	5 (3.51–6.62)	7,018 (4,841–9,907)	5.41 (4.28–6.78)	9,183 (6,701–12,326)	5.53 (4.44–6.9)	10,833 (8,451–13,875)	5.48 (4.49–6.77)	0.32 (0.26–0.37)
Lip and oral cavity cancer	10,226 (8,703–12,032)	16.6 (14.02–19.55)	15,729 (13,741–18,051)	20.97 (18.26–23.92)	20,764 (17,278–24,969)	21.29 (17.8–25.43)	28,579 (22,907–35,935)	21.93 (17.83–27.56)	1.00 (0.97–1.04)
Liver cancer	1,976 (1,345–2,622)	3.27 (2.16–4.42)	2,556 (2,020–3,110)	3.63 (2.8–4.5)	3,160 (2,515–3,825)	3.52 (2.73–4.32)	3,884 (3,101–4,736)	3.27 (2.56–4.02)	0 (−0.01 to −0.01)
Malignant skin melanoma	263 (198–341)	0.41 (0.32–0.54)	379 (257–474)	0.48 (0.35–0.6)	512 (337–639)	0.49 (0.35–0.62)	732 (486–960)	0.53 (0.36–0.68)	0.88 (0.85–0.92)
Mesothelioma	88 (47–162)	0.15 (0.08–0.28)	136 (77–232)	0.19 (0.11–0.31)	177 (107–287)	0.2 (0.12–0.31)	237 (146–379)	0.2 (0.13–0.31)	1.07 (1.04–1.09)
Multiple myeloma	759 (614–939)	1.34 (1.06–1.64)	1,037 (837–1,220)	1.59 (1.27–1.87)	1,347 (1,056–1,664)	1.63 (1.27–1.99)	1,820 (1,354–2,264)	1.65 (1.21–2.05)	0.72 (0.7–0.75)
Nasopharynx cancer	944 (804–1,076)	1.44 (1.22–1.66)	1,283 (1,111–1,502)	1.58 (1.36–1.85)	1,600 (1,317–1,942)	1.49 (1.22–1.8)	2,012 (1,601–2,497)	1.4 (1.12–1.75)	−0.08 (−0.1 to −0.05)
Non-Hodgkin lymphoma	931 (662–1,224)	1.31 (0.96–1.68)	1,537 (1,005–2,156)	1.73 (1.18–2.36)	2,307 (1,464–3,379)	1.93 (1.29–2.77)	3,258 (2,185–4,629)	2.05 (1.41–2.87)	1.57 (1.54–1.63)
Non-melanoma skin cancer (basal-cell carcinoma)	473 (366–637)	0.62 (0.49–0.84)	614 (474–824)	0.63 (0.5–0.84)	835 (630–1,126)	0.63 (0.48–0.84)	1,133 (850–1,552)	0.63 (0.48–0.85)	2.23 (2.2–2.27)
Non-melanoma skin cancer (squamous-cell carcinoma)	204 (170–242)	0.4 (0.34–0.47)	227 (192–263)	0.4 (0.35–0.47)	270 (227–315)	0.4 (0.34–0.46)	335 (280–397)	0.37 (0.32–0.44)	0.03 (0.03–0.04)
Other leukemia	2,460 (845–4,771)	1.72 (0.75–2.93)	2,482 (1,111–4,422)	1.52 (0.83–2.39)	3,007 (1,560–5,062)	1.47 (0.88–2.24)	3,235 (1,861–5,176)	1.39 (0.89–2.03)	−0.25 (−0.26 to −0.24)
Other pharynx cancer	1,872 (1,557–2,216)	3.12 (2.59–3.71)	2,853 (2,480–3,307)	3.94 (3.43–4.55)	3,696 (2,976–4,525)	3.93 (3.2–4.81)	4,947 (3,807–6,429)	3.93 (3.05–5.04)	−0.73 (−0.84 to −0.65)
Ovarian cancer	1,915 (1,481–2,418)	2.94 (2.29–3.74)	3,265 (2,515–3,979)	4.09 (3.32–4.98)	6,134 (4,027–8,481)	5.86 (3.99–8.01)	10,783 (5,511–17,344)	7.7 (4.12–12.33)	0.81 (0.79–0.84)
Pancreatic cancer	815 (668–958)	1.47 (1.2–1.73)	1,274 (1,112–1,449)	2.02 (1.75–2.29)	2,023 (1,674–2,449)	2.57 (2.1–3.11)	3,033 (2,376–3,955)	2.91 (2.27–3.76)	3.39 (3.36–3.43)
Prostate cancer	2,764 (1,986–3,764)	5.56 (4.02–7.71)	3,377 (2,512–4,013)	6.35 (4.81–7.61)	4,196 (3,058–5,397)	6.47 (4.85–8.22)	5,973 (4,203–7,972)	6.85 (4.99–8.99)	2.4 (2.36–2.45)
Stomach cancer	3,695 (2,960–4,340)	6.58 (5.23–7.74)	5,166 (4,489–5,885)	7.85 (6.78–8.98)	6,048 (5,035–7,113)	7.32 (6.14–8.62)	7,057 (5,827–8,596)	6.45 (5.34–7.79)	0.7 (0.66–0.73)
Testicular cancer	575 (399–1,051)	0.53 (0.4–0.79)	938 (662–1,613)	0.68 (0.51–1)	1,551 (983–2,854)	0.85 (0.56–1.42)	2,499 (1,494–4,460)	1.1 (0.68–1.87)	−0.04 (−0.07 to 0)
Thyroid cancer	1,121 (943–1,338)	1.55 (1.32–1.83)	2,038 (1,645–2,414)	2.23 (1.85–2.57)	3,152 (2,465–3,900)	2.57 (2.08–3.11)	4,885 (3,650–6,521)	2.96 (2.3–3.81)	2.54 (2.47–2.62)
Tracheal, bronchus, and lung cancer	7,904 (6,635–9,210)	13.92 (11.67–16.27)	11,708 (10,010–13,610)	17.67 (15.19–20.46)	14,621 (11,540–18,092)	17.38 (13.79–21.4)	18,401 (13,970–24,265)	16.43 (12.47–21.49)	2.25 (2.19–2.32)
Uterine cancer	1,299 (1,042–1,738)	2.21 (1.75–2.98)	2,232 (1,782–2,751)	3.19 (2.53–3.99)	3,300 (2,435–4,244)	3.68 (2.76–4.76)	5,011 (3,575–7,055)	4.16 (3–5.76)	0.57 (0.54–0.6)
**Geographical area**									
Pakistan	1,283,332 (1,064,900–1,543,308)	1,566.48 (1,299.55–1,888.55)	1,632,382 (1,355,875–1,965,871)	1,599.8 (1,327.83–1,917.91)	2,142,645 (1,769,346–2,588,666)	1,608.84 (1,336.63–1,930.71)	2,769,507 (2,297,654–3,337,263)	1,617.33 (1,349.92–1,930.3)	0.11 (0.11–0.12)
Sindh	271,165 (223,111–328,962)	1,550.44 (1,281.33–1,871.94)	354,175 (291,408–430,729)	1,596.54 (1,324.55–1,925.67)	471,409 (389,733–571,252)	1,603.98 (1,323.64–1,928.4)	609,543 (500,297–737,306)	1,609.73 (1,338.02–1,932.39)	0.13 (0.13–0.14)
Punjab	717,974 (598,034–859,769)	1,581.28 (1,315.96–1,908.59)	906,109 (754,517–1,086,699)	1,610.48 (1,348.66–1,933.94)	1,173,228 (976,136–1,413,203)	1,619.25 (1,351.49–1,948.63)	1,489,130 (1,243,110–1,799,634)	1,630.1 (1,363.4–1,941.04)	0.11 (0.1–0.11)
Khyber Pakhtunkhwa	189,874 (156,618–230,256)	1,534.62 (1,266.92–1,862.11)	240,387 (199,694–290,833)	1,559.68 (1,301.67–1,878.48)	318,934 (264,113–387,179)	1,571.33 (1,299.79–1,892.88)	423,192 (350,577–513,908)	1,577.97 (1,315.64–1,903.79)	0.1 (0.09–0.1)
Islamabad Capital Territory	6,084 (5,020–7,369)	1,576.73 (1,309.79–1,909.33)	9,877 (8,170–11,939)	1,635.74 (1,360.16–1,968.09)	16,658 (13,644–20,374)	1,667.65 (1,390.71–1,999.67)	26,339 (21,541–32,410)	1,684.44 (1,404.21–2,019.92)	0.23 (0.22–0.23)
Gilgit-Baltistan	8,378 (6,903–10,127)	1,536.67 (1,262.97–1,850.72)	11,731 (9,703–14,173)	1,574.98 (1,304.6–1,894.77)	16,712 (13,844–20,251)	1,579.15 (1,310.39–1,904.61)	23,344 (19,330–28,283)	1,580.05 (1,315.95–1,897.99)	0.1 (0.09–0.1)
Baluchistan	60,876 (50,333–73,606)	1,554.76 (1,287.49–1,879.47)	73,811 (61,144–89,087)	1,609.52 (1,340.63–1,932.63)	100,349 (82,865–121,377)	1,619.76 (1,352.13–1,943.75)	140,691 (116,310–169,735)	1,621.67 (1,349–1,942.46)	0.15 (0.14–0.15)
Azad Jammu & Kashmir	28,981 (24,110–34,702)	1,570.46 (1,297.74–1,889.38)	36,292 (30,050–43,746)	1,614.22 (1,344.9–1,942.49)	45,355 (37,521–55,061)	1,624.63 (1,351.37–1,957.98)	57,267 (47,255–69,606)	1,632.4 (1,364.59–1,944.32)	0.13 (0.13–0.14)
**Gender**									
Male	627,752 (521,714–760,293)	1,505.07 (1,242.56–1,863.44)	781,978 (647,388–948,956)	1,536.95 (1,277.09–1,890.06)	1,008,566 (833,234–1,226,885)	1,540.79 (1,270.74–1,899.27)	1,289,617 (1,067,065–1,569,033)	1,540.98 (1,276.06–1,899.89)	0.09 (0.08–0.09)
Female	655,580 (542,659–792,828)	1,622.76 (1,343.77–1,953.58)	850,404 (703,044–1,032,853)	1,661.5 (1379.54–2,002.36)	1,134,079 (936,487–1,372,719)	1,677.74 (1,398.1–2,013.78)	1,479,890 (1,220,322–1,803,182)	1,694.51 (1,413.7–2,033.82)	0.15 (0.15–0.16)
**Total**	1,283,332		1,632,382		2,142,645		2,769,507		

ASR = Age-Standardised Rate; AAPC: Average Annual Percent Change; CI: Confidence Interval; UI: Uncertainty Interval

**Table 2.** Cancer mortality trends by type, gender and province (1990–2019).

Mortality	1990		2000		2010		2019		AAPC between 1990 and 2019 (95% CI)
	Cases (95% UI)	ASR/100,000 (95% UI)	Cases (95% UI)	ASR/100,000 (95% UI)	Cases (95% UI)	ASR/100,000 (95% UI)	Cases (95% UI)	ASR/100,000 (95% UI)	
**Cancer subtype**									
Acute lymphoid leukemia	699 (411–1,366)	0.5 (0.33–0.85)	1,048 (696–1,765)	0.6 (0.43–0.93)	1,397 (1,006–2,009)	0.63 (0.46–0.88)	1,707 (1,281–2,314)	0.65 (0.49–0.87)	0.96 (0.87–1.05)
Acute myeloid leukemia	605 (471–822)	0.68 (0.53–0.96)	873 (663–1,138)	0.8 (0.65–0.99)	1,188 (920–1,487)	0.85 (0.67–1.06)	1,576 (1,230–1,965)	0.9 (0.7–1.13)	0.96 (0.91–0.99)
Bladder cancer	2,806 (2,277–3,393)	5.48 (4.42–6.64)	3,544 (3,018–4,111)	6.29 (5.34–7.33)	4,469 (3,607–5,351)	6.52 (5.26–7.81)	5,818 (4,576–7,412)	6.42 (5.03–8.09)	0.54 (0.52–0.57)
Brain and central nervous system cancer	1,784 (1,273–2,596)	1.87 (1.44–2.54)	2,489 (1,751–3,346)	2.21 (1.64–2.82)	3,225 (2,253–4,088)	2.22 (1.64–2.81)	4,105 (2,847–5,344)	2.27 (1.6–2.98)	0.67 (0.62–0.71)
Breast cancer	9,807 (7,126–13,283)	16.19 (11.69–22.13)	16,107 (13,254–19,604)	22.15 (18.04–27.38)	22,607 (17,763–27,913)	24.53 (19.33–30.07)	32,118 (24,388–43,374)	26.34 (20.2–34.86)	1.71 (1.69–1.75)
Cervical cancer	1,349 (1,087–1,692)	2.13 (1.7–2.66)	2,068 (1,589–2,506)	2.66 (2.07–3.24)	2,416 (1,823–3,125)	2.4 (1.82–3.04)	2,947 (2,095–4,139)	2.22 (1.57–3.06)	0.15 (0.12–0.17)
Chronic lymphoid leukemia	410 (336–512)	0.75 (0.61–0.93)	592 (493–704)	0.96 (0.79–1.15)	760 (621–936)	0.99 (0.82–1.23)	1,002 (805–1,269)	0.99 (0.8–1.24)	0.94 (0.92–0.97)
Chronic myeloid leukemia	766 (489–1,263)	0.9 (0.7–1.16)	944 (654–1,380)	0.97 (0.76–1.24)	1,009 (710–1,396)	0.84 (0.62–1.08)	1,107 (845–1,447)	0.72 (0.55–0.96)	−0.77 (−0.79 to −0.74)
Colon and rectum cancer	2,968 (2,546–3,430)	5.43 (4.66–6.3)	4,715 (4,132–5,330)	7.49 (6.52–8.48)	6,271 (5,211–7,433)	8.03 (6.71–9.51)	8,605 (6,927–10,776)	8.34 (6.72–10.41)	1.49 (1.47–1.52)
Esophageal cancer	4,128 (3,485–4,824)	7.34 (6.2–8.63)	5,826 (4,911–6,576)	8.93 (7.57–10.06)	7,159 (5,889–8,523)	8.73 (7.23–10.33)	8,953 (7,184–11,011)	8.23 (6.62–9.96)	0.41 (0.39–0.44)
Gallbladder and biliary tract cancer	1,604 (1,297–2,066)	2.93 (2.35–3.81)	2,441 (2,003–2,914)	3.9 (3.18–4.71)	2,912 (2,370–3,516)	3.77 (3.08–4.6)	3,681 (2,898–4,607)	3.61 (2.89–4.49)	0.71 (0.69–0.72)
Hodgkin lymphoma	1,152 (875–1,486)	1.45 (1.1–1.92)	1,629 (1,197–2,034)	1.6 (1.23–2.08)	1,947 (1,399–2,447)	1.42 (1.05–1.85)	2,270 (1,645–3,003)	1.28 (0.94–1.72)	−0.41 (−0.44 to −0.39)
Kidney cancer	398 (327–478)	0.66 (0.54–0.79)	643 (552–759)	0.93 (0.8–1.1)	934 (755–1,155)	1.08 (0.88–1.34)	1,314 (992–1,763)	1.16 (0.88–1.56)	1.95 (1.92–1.98)
Larynx cancer	3,428 (2,853–4,086)	5.99 (4.98–7.12)	4,824 (4,105–5,631)	7.17 (6.09–8.36)	5,494 (4,394–6,755)	6.4 (5.19–7.81)	6,565 (5,042–8,600)	5.75 (4.47–7.44)	−0.11 (−0.14 to −0.08)
Leukemia	3,364 (2,350–4,511)	3.76 (2.94–4.55)	4,439 (3,456–5,711)	4.23 (3.53–5.02)	5,359 (4,319–6,577)	4.07 (3.42–4.9)	6,492 (5,336–7,817)	3.94 (3.28–4.72)	0.18 (0.13–0.21)
Lip and oral cavity cancer	7,141 (6,092–8,406)	12.18 (10.34–14.32)	10,726 (9,303–12,253)	15.26 (13.17–17.38)	13,459 (11,296–16,088)	14.99 (12.56–17.86)	17,567 (14,062–22,169)	14.72 (11.9–18.34)	0.68 (0.65–0.7)
Liver cancer	2,060 (1,428–2,752)	3.47 (2.31–4.72)	2,615 (2,074–3,209)	3.83 (2.94–4.79)	3,198 (2,538–3,856)	3.72 (2.88–4.55)	3,912 (3,165–4,833)	3.46 (2.75–4.3)	0 (–0.02–0.03)
Malignant skin melanoma	231 (178–303)	0.38 (0.3–0.52)	324 (228–399)	0.45 (0.34–0.57)	405 (277–499)	0.44 (0.32–0.56)	533 (350–691)	0.43 (0.3–0.57)	0.44 (0.42–0.45)
Mesothelioma	67 (36–126)	0.11 (0.06–0.22)	104 (61–176)	0.15 (0.09–0.25)	136 (85–220)	0.16 (0.1–0.25)	183 (113–286)	0.16 (0.1–0.25)	1.21 (1.18–1.23)
Multiple myeloma	722 (571–901)	1.31 (1.02–1.62)	969 (775–1,136)	1.54 (1.22–1.81)	1,225 (948–1,487)	1.55 (1.17–1.88)	1,618 (1,192–2,027)	1.54 (1.11–1.91)	0.59 (0.58–0.61)
Nasopharynx cancer	906 (772–1,036)	1.42 (1.21–1.64)	1,214 (1,041–1,404)	1.55 (1.32–1.79)	1,495 (1,232–1,789)	1.45 (1.2–1.73)	1,859 (1,481–2,325)	1.35 (1.08–1.7)	−0.15 (−0.18 to −0.13)
Non-Hodgkin lymphoma	984 (674–1,293)	1.42 (1.04–1.84)	1,525 (993–2,120)	1.83 (1.26–2.49)	2,095 (1,321–3,059)	1.97 (1.32–2.83)	2,756 (1,854–3,986)	1.99 (1.4–2.85)	1.19 (1.16–1.21)
Non-melanoma skin cancer (basal-cell carcinoma)	-----	-----	-----	-----	-----	-----	-----	-----	-----
Non-melanoma skin cancer (squamous-cell carcinoma)	178 (113–246)	0.37 (0.23–0.51)	249 (178–345)	0.44 (0.30–0.62)	311 (233–425)	0.45 (0.33–0.61)	410 (310–572)	0.46 (0.34–0.63)	1.22 (1.01 to −1.56)
Other leukemia	884 (411–1,452)	0.92 (0.51–1.29)	982 (564–1,519)	0.91 (0.58–1.23)	1,004 (685–1,426)	0.77 (0.55–1.04)	1,099 (790–1,500)	0.69 (0.51–0.93)	−0.99 (−1.01 to −0.96)
Other pharynx cancer	1,838 (1,535–2,219)	3.11 (2.59–3.77)	2,759 (2,376–3,207)	3.9 (3.36–4.52)	3,511 (2,834–4,319)	3.85 (3.13–4.72)	4,612 (3,541–6,024)	3.79 (2.95–4.91)	0.69 (0.67–0.71)
Ovarian cancer	1,420 (1,104–1,762)	2.34 (1.81–2.98)	2,322 (1,836–2,845)	3.23 (2.64–3.96)	4,148 (2,757–5,713)	4.52 (3.07–6.23)	7,002 (3,672–11,477)	5.75 (3.03–9.42)	3.16 (3.12–3.18)
Pancreatic cancer	859 (716–1,006)	1.58 (1.31–1.86)	1,321 (1,150–1,508)	2.16 (1.87–2.47)	2,077 (1,697–2,497)	2.75 (2.26–3.29)	3,101 (2,404–3,982)	3.1 (2.41–3.96)	2.37 (2.33–2.42)
Prostate cancer	2,840 (2,048–3,910)	5.93 (4.27–8.28)	3,319 (2,490–3,960)	6.62 (5.08–7.99)	3,761 (2,785–4,743)	6.36 (4.87–8.1)	4,922 (3,612–6,477)	6.29 (4.75–8.27)	0.21 (0.18–0.24)
Stomach cancer	3,885 (3,135–4,574)	7.13 (5.66–8.46)	5,296 (4,608–5,996)	8.4 (7.24–9.52)	6,127 (5,140–7,147)	7.83 (6.56–9.1)	7,105 (5,879–8,688)	6.87 (5.71–8.31)	−0.11 (−0.13 to −0.09)
Testicular cancer	268 (221–326)	0.3 (0.24–0.37)	434 (355–530)	0.38 (0.31–0.46)	589 (436–772)	0.38 (0.28–0.5)	797 (568–1,113)	0.4 (0.29–0.56)	0.98 (0.94–1.02)
Thyroid cancer	500 (432–599)	0.82 (0.71–1.01)	808 (696–923)	1.11 (0.97–1.27)	1,030 (869–1,214)	1.12 (0.95–1.31)	1,317 (1,077–1,610)	1.08 (0.9–1.32)	0.98 (0.96–1)
Tracheal, bronchus, and lung cancer	8,230 (6,953–9,625)	14.75 (12.42–17.27)	11,962 (10,214–13,917)	18.54 (15.85–21.53)	14,819 (11,510–18,507)	18.22 (14.31–22.58)	18,550 (14,209–23,969)	17.16 (13.14–22.05)	0.52 (0.49–0.54)
Uterine cancer	865 (688–1,179)	1.55 (1.22–2.16)	1,405 (1,122–1,731)	2.18 (1.75–2.71)	1,858 (1,414–2,368)	2.33 (1.8–3.01)	2,491 (1,826–3,412)	2.36 (1.77–3.21)	1.48 (1.46–1.5)
**Geographical area**									
Pakistan	73,424 (64,996–80,969)	122.16 (107.34–135.18)	106,261 (94,887–116,454)	151.36 (135.22–165.49)	136,615 (118,158–157,595)	153.96 (133.45–176.83)	179,773 (152,043–214,194)	153.52 (131.06–181.75)	0.79 (0.78–0.81)
Sindh	12,428 (10,052–15,061)	110.3 (88.55–133.32)	20,179 (16,085–24,586)	147.51 (118.97–177.76)	26,430 (20,985–33,299)	147.12 (119.3–183.2)	35,072 (27,488–44,755)	143.65 (113.61–182.8)	0.93 (0.91–0.96)
Punjab	47,078 (41,032–53,144)	132.51 (115.42–149.31)	64,662 (56,273–72,722)	159.53 (138.15–179.46)	82,017 (68,572–96,877)	162.34 (137.63–190.79)	107,232 (87,374–130,264)	162.94 (134.63–196.83)	0.73 (0.71–0.75)
Khyber Pakhtunkhwa	8,550 (6,270–10,946)	98.85 (73.05–126.2)	13,101 (10,153–16,168)	123.81 (95.56–151.62)	17,558 (13,788–21,936)	131.11 (102.83–163.19)	23,120 (17,850–29,570)	132.3 (101.47–167.34)	1.01 (0.99–1.03)
Islamabad Capital Territory	290 (209–366)	119.41 (88.9–149.42)	559 (435–694)	155.32 (122–191.91)	874 (669–1,112)	161.34 (125.62–199)	1,310 (994–1,692)	155.9 (120.12–195.37)	0.92 (0.89–0.96)
Gilgit-Baltistan	347 (245–462)	101.95 (75.25–131.63)	629 (485–791)	134.9 (105.56–167.79)	859 (647–1,120)	134.87 (102.2–173.08)	1,168 (877–1,535)	132.75 (99.47–174.91)	0.91 (0.89–0.94)
Balochistan	3,072 (2,018–3,903)	117.94 (84.11–147.68)	4,664 (3,666–5,876)	158.86 (125.81–196.77)	5,899 (4,606–7,506)	159.79 (125.57–199.35)	8,023 (6,161–10,410)	156.86 (121.87–203.02)	1.01 (0.97–1.06)
Azad Jammu & Kashmir	1,658 (1,342–2,051)	117.87 (95.3–144.66)	2,466 (1,946–3,045)	152.73 (121.46–187.35)	2,977 (2,320–3,833)	155.67 (122.37–198.12)	3,848 (2,938–4,955)	157.1 (119.83–199.76)	1 (0.98–1.02)
**Gender**									
Male	41,079 (34,844–47,478)	126.17 (107.24–145.09)	56,893 (48,378–66,057)	155.03 (132.31–178.95)	70,044 (55,576–85,671)	154.6 (123.3–187.73)	88,241 (68,915–113,114)	149.4 (115.99–190.82)	0.6 (0.58–0.62)
Female	32,345 (27,438–37,740)	116.42 (97.63–137.65)	49,368 (42,995–55,758)	146.79 (126.59–165.13)	66,571 (54,883–79,448)	153.4 (126.83–182.1)	91,533 (72,050–117,874)	158.04 (125.82–199.62)	1.07 (1.05–1.09)
**Total**	73,424		106,261		136,615		179,773		

**Table 3.** DALY trends for cancers by type, gender and province (1990–2019).

DALYs	1990		2000		2010		2019		AAPC between 1990 and 2019 (95% CI)
	Cases (95% UI)	ASR/100,000 (95% UI)	Cases (95% UI)	ASR/100,000 (95% UI)	Cases (95% UI)	ASR/100,000 (95% UI)	Cases (95% UI)	ASR/100,000 (95% UI)	
**Cancer subtype**									
Acute lymphoid leukemia	52,913 (29,445–107,459)	33.56 (20.26–63.32)	79,057 (50,412–137,802)	40.95 (27.61–67.32)	105,241 (74,371–154,819)	43.01 (31.21–61.67)	127,533 (93,911–175,502)	45.07 (33.56–61.62)	3.15 (2.94–3.33)
Acute myeloid leukemia	34,235 (24,347–52,244)	29.19 (22.9–38.58)	50,750 (35,333–72,268)	34.76 (26.72–44.72)	69,420 (50,571–89,775)	37.34 (28.99–46.69)	90,151 (67,285–116,288)	40.05 (31.26–50.17)	3.43 (3.34–3.52)
Bladder cancer	61,139 (49,708–73,023)	109.48 (88.37–131.19)	82,347 (70,817–95,039)	127.51 (109.57–147.61)	107,072 (86,734–129,235)	131.55 (107–157.67)	139,936 (110,285–178,027)	129.29 (102.16–164.53)	2.9 (2.88–2.92)
Brain and central nervous system cancer	107,535 (69,104–170,537)	86.05 (62.68–124.58)	146,689 (96,828–210,688)	99.27 (70.43–132.65)	191,150 (127,521–246,505)	101.25 (71.11–127.47)	237,760 (161,752–309,213)	105.47 (72.77–137.31)	2.84 (2.75–2.93)
Breast cancer	325,816 (239,525–433,840)	492.19 (360.72–660.87)	558,810 (463,595–666,625)	680.4 (559.98–824.85)	788,356 (617,517–985,305)	733.76 (578.57–905.46)	1,119,378 (847,412–1,520,925)	783.3 (597.3–1,054.53)	4.38 (4.35–4.42)
Cervical cancer	48,396 (39,053–60,790)	70.9 (57.13–89.6)	77,330 (59,093–94,344)	89.66 (69.01–109.04)	91,182 (67,918–119,158)	79.68 (60.12–102.99)	110,786 (78,203–158,090)	73.05 (52.23–102.66)	2.91 (2.88–2.94)
Chronic lymphoid leukemia	10,556 (8,653–13,072)	17.64 (14.49–21.87)	16,097 (13,462–19,232)	22.65 (18.95–27)	21,023 (16,909–25,926)	23.16 (18.86–28.58)	27,827 (21,960–35,968)	22.99 (18.43–29.18)	3.4 (3.38–3.43)
Chronic myeloid leukemia	41,818 (20,055–86,132)	36.54 (24.01–58.51)	49,378 (29,551–84,465)	37.75 (26.9–53.47)	51,387 (33,405–78,919)	31.92 (22.65–43.72)	54,960 (40,157–76,659)	27.79 (21.05–36.46)	0.99 (0.9–1.07)
Colon and rectum cancer	77,682 (66,197–89,591)	127.68 (109.09–147.26)	133,571 (116,797–151,591)	181.75 (158.91–205.35)	181,777 (150,972–216,924)	192.06 (160.07–227.44)	249,316 (199,384–312,981)	198.31 (159.17–248.72)	4.11 (4.09–4.14)
Esophageal cancer	112,246 (95,366–131,766)	185.61 (157.4–216.94)	167,010 (141,944–188,794)	229.15 (193.3–258.95)	208,464 (170,019–248,539)	221.22 (181.92–263.45)	260,192 (204,466–323,900)	207.08 (165.9–255.39)	2.97 (2.94–3.02)
Gallbladder and biliary tract cancer	40,914 (33,942–52,082)	68.96 (56.57–88.1)	66,236 (54,532–77,235)	93.4 (77.11–109.51)	79,863 (64,511–95,782)	88.26 (71.58–105.73)	100,554 (78,212–127,934)	83.66 (65.6–104.73)	3.15 (3.12–3.18)
Hodgkin lymphoma	57,459 (41,974–74,380)	60.23 (45.53–77.24)	85,412 (59,691–107,529)	68.88 (50.41–85.93)	105,391 (72,629–133,887)	63.04 (45.26–79.08)	122,851 (86,562–161,684)	57.51 (41.62–75.57)	2.67 (2.61–2.72)
Kidney cancer	13,110 (10,753–16,621)	17.58 (14.49–21.21)	21,602 (17,570–26,277)	24.86 (21.41–29.31)	31,826 (25,202–39,663)	28.75 (23.26–35.71)	44,131 (33,808–58,932)	30.95 (23.5–41.47)	4.33 (4.27–4.41)
Larynx cancer	97,326 (81,027–116,131)	159.62 (132.98–190.39)	143,876 (122,546–167,576)	194.59 (165.59–226.27)	167,204 (132,804–206,065)	173.34 (138.54–213.09)	200,054 (153,489–264,943)	155.08 (119.63–203.66)	2.55 (2.52–2.59)
Leukemia	195,066 (118,387–293,639)	157.38 (112.13–209.06)	254,966 (181,729–356,229)	173.82 (137.71–220.96)	306,782 (233,892–394,884)	166.83 (134.57–204.08)	363,565 (290,877–449,774)	164.17 (134.44–198.88)	2.21 (2.1–2.3)
Lip and oral cavity cancer	222,000 (188,451–262,436)	343.14 (293.41–405.54)	354,194 (307,565–405,176)	441.29 (382.48–504.58)	453,821 (378,474–543,346)	430.09 (361.61–513.01)	594,089 (471,317–754,897)	421.87 (338.19–535.02)	3.45 (3.42–3.48)
Liver cancer	64,587 (47,309–84,080)	91.77 (63.93–122.11)	85,075 (69,774–102,029)	101.48 (80.91–124.33)	106,355 (87,421–125,805)	98.05 (78.27–118.19)	129,034 (104,785–157,625)	91.09 (73.72–112.15)	2.45 (2.42–2.49)
Malignant skin melanoma	7,829 (5,670–10,053)	11.06 (8.18–14.02)	11,706 (7,579–14,845)	13.21 (8.83–16.36)	15,103 (9,622–18,813)	12.89 (8.58–16)	19,920 (12,886–26,235)	12.87 (8.37–16.85)	3.27 (3.24–3.3)
Mesothelioma	2,054 (1,064–3,589)	3.15 (1.67–5.63)	3,333 (1,845–5,970)	4.18 (2.41–7.2)	4,416 (2,613–7,400)	4.28 (2.64–6.95)	5,904 (3,503–9,531)	4.32 (2.64–6.78)	3.72 (3.69–3.74)
Multiple myeloma	18,661 (15,288–23,349)	31.37 (25.5–39.4)	26,423 (21,203–31,382)	37.34 (29.8–43.92)	34,184 (27,351–41,880)	37.49 (29.39–45.71)	45,287 (33,714–57,255)	37.23 (27.61–46.49)	3.11 (3.08–3.14)
Nasopharynx cancer	32,396 (27,566–37,064)	46.51 (39.57–53.31)	45,741 (39,388–52,862)	51.8 (44.4–59.9)	57,538 (47,469–68,683)	48.74 (40.17–58.39)	71,647 (57,194–88,599)	45.65 (36.3–57.03)	2.79 (2.76–2.82)
Non-Hodgkin lymphoma	41,518 (25,573–58,531)	47.02 (31.63–62.24)	67,065 (40,191–96,740)	61.32 (39.24–85.45)	93,632 (55,664–138,747)	65.97 (41.25–96.76)	122,027 (77,460–178,060)	67.09 (44.73–96.9)	3.83 (3.76–3.91)
Non-melanoma skin cancer (basal-cell carcinoma)	0 (0–1)	0 (0–0)	0 (0–1)	0 (0–0)	0 (0–1)	0 (0–0)	1 (0–1)	0 (0–0)	3.05 (3.05–3.06)
Non-melanoma skin cancer (squamous-cell carcinoma)	4,451 (2,494–6,033)	7.88 (4.3–10.72)	5,386 (3,903–6,827)	8.23 (5.71–10.41)	6,551 (4,899–8,431)	7.88 (5.78–10.07)	8,461 (6,470–10,953)	7.65 (5.68–9.87)	2.23 (2.21–2.26)
Other leukemia	55,545 (21,606–102,315)	40.44 (19.22–66.14)	59,682 (30,399–103,529)	37.71 (22.21–57.3)	59,712 (36,841–92,201)	31.39 (21.55–44.93)	63,093 (42,445–93,641)	28.27 (20.15–39.41)	0.48 (0.38–0.57)
Other pharynx cancer	55,836 (46,823–66,945)	89.28 (74.67–107.28)	87,425 (75,528–102,164)	113.56 (97.76–132.4)	113,016 (91,181–139,624)	111.86 (90.43–138.18)	148,444 (114,299–193,527)	109.78 (84.18–143.17)	3.45 (3.42–3.48)
Ovarian cancer	46,538 (35,377–57,205)	70.03 (54.01–86.55)	78,814 (57,332–97,120)	96.28 (73.52–118.43)	142,832 (93,099–197,498)	132.98 (88.59–183.56)	241,404 (122,744–390,862)	168.77 (88.55–272.27)	5.85 (5.81–5.88)
Pancreatic cancer	20,993 (17,490–24,304)	35.74 (29.84–41.53)	34,130 (29,742–38,993)	49.38 (42.99–56.3)	54,812 (44,649–66,104)	62.05 (50.57–74.82)	81,818 (63,158–105,869)	69.62 (54.01–89.47)	4.87 (4.81–4.93)
Prostate cancer	50,396 (36,066–67,442)	97.18 (70.1–131.84)	61,536 (45,036–73,236)	109.08 (80.99–130.05)	72,654 (51,906–93,000)	105.21 (77.33–132.82)	95,910 (68,727–127,158)	104.52 (76.74–136.75)	2.23 (2.2–2.27)
Stomach cancer	100,449 (82,664–117,355)	165.67 (134.8–193.87)	149,096 (130,092–168,468)	203.33 (176.76–230.39)	176,558 (146,456–207,219)	186.66 (155.83–218.04)	204,475 (166,415–252,396)	162.7 (133.94–198.78)	2.5 (2.48–2.53)
Testicular cancer	15,079 (12,454–18,471)	15.46 (12.55–18.87)	24,700 (20,058–30,491)	19.3 (15.69–23.5)	33,813 (25,146–44,678)	19.89 (14.72–26.14)	45,891 (32,596–64,455)	21.06 (14.99–29.52)	3.9 (3.86–3.94)
Thyroid cancer	16,908 (14,563–19,904)	23.94 (20.77–28.23)	29,276 (24,423–33,728)	33.09 (28.17–37.62)	38,327 (31,722–45,824)	33.09 (27.77–39.04)	49,409 (39,271–60,858)	32.29 (26.35–39.3)	3.78 (3.75–3.81)
Tracheal, bronchus, and lung cancer	214,427 (181,841–248,653)	361.3 (305.47–420.49)	329,901 (280,892–383,936)	464.18 (396.05–540.62)	416,861 (321,808–524,148)	453.8 (350.78–567.63)	522,648 (400,317–680,663)	425.72 (327.1–550.78)	3.12 (3.08–3.15)
Uterine cancer	23,279 (18,943–30,326)	38.76 (31.29–51.64)	39,787 (31,321–48,984)	55.23 (44.04–68.79)	53,056 (39,595–67,954)	57.58 (43.88–73.33)	71,084 (50,858–96,356)	58.1 (43–79.74)	3.93 (3.9–3.97)
**Geographical area**									
Pakistan	2,414,828 (2,105,713–2,713,915)	3,363.75 (3,000.28–3,703.96)	3,639,168 (3,254,522–4,068,820)	4,224.26 (3,778.87–4,628.45)	4,778,563 (4,102,144–5,528,207)	4,270.8 (3,693.01–4,935.43)	6,268,083 (5,269,382–7,399,739)	3,439.9 (2,871.7–4,122.04)	0.85 (0.82–0.88)
Sindh	412,712 (328,106–499,818)	2,957.09 (2,373.6–3,596.82)	714,569 (570,005–871,910)	4,054.54 (3,246.45–4,955.59)	944,447 (741,185–1,181,726)	4,011.3 (3,191.94–5,042.02)	1,221,334 (944,552–1,553,931)	3,330.1 (2,540.4–4,239.04)	0.99 (0.96–1.03)
Punjab	1,548,329 (1,336,942–1,781,591)	3,710.68 (3,252.72–4,189.82)	2,200,970 (1,878,003–2,523,407)	4,485.13 (3,904.54–5,071.07)	2,853,279 (2,365,471–3,395,420)	4,542.35 (3,784.06–5,384.21)	3,717,719 (2,978,642–4,505,382)	3,538.2 (2,855.7–4,316.5)	0.75 (0.73–0.77)
Khyber Pakhtunkhwa	274,439 (198,997–352,757)	2,700.42 (1,969.82–3,471.02)	432,515 (337,452–536,418)	3,458.43 (2,689.88–4,296.31)	596,704 (470,273–744,613)	3,640.96 (2,845.62–4,569.96)	796,705 (626,009–1,020,083)	3,239.2 (2,474.3–4,209.5)	1.07 (1.05–1.1)
Islamabad Capital Territory	9,329 (6,195–11,926)	3,042.17 (2,150.54–3,866.46)	18,133 (14,105–22,468)	3,970.52 (3,092.71–4,929.98)	28,721 (21,936–37,054)	4,043.23 (3,114.16–5,144.03)	44,480 (32,780–58,729)	3,053.8 (2,116.2–3,968.3)	0.91 (0.87–0.95)
Gilgit-Baltistan	12,500 (8,044–17,185)	2,839.69 (2,009.65–3,773.29)	23,099 (17,683–29,091)	3,866.88 (2,991.95–4,858.32)	32,159 (24,165–41,676)	3,817.19 (2,868.72–4,956.81)	43,960 (32,632–56,773)	3,440.3 (2,513.5–4,557.9)	0.96 (0.92–1.01)
Balochistan	105,033 (61,585–134,814)	3,248.15 (2,067.81–4,117.22)	168,674 (131,570–213,844)	4,478.91 (3,501.89–5,648.68)	224,774 (174,872–286,768)	4,492.57 (3,484.52–5,721.2)	318,498 (244,064–403,820)	3,552.41 (2,636.6–4,685.9)	1.12 (1.08–1.17)
Azad Jammu & Kashmir	52,486 (41,918–65,159)	3,236.5 (2,599.24–4,016.64)	81,208 (63,723–101,009)	4,256.25 (3,321.24–5,308.68)	98,480 (76,028–126,715)	4,241.82 (3,286–5,478.07)	125,386 (93,958–162,886)	3,615.4 (2,774.1–4,890.4)	0.93 (0.91–0.95)
Gender									
Male	1,302,256 (1,070,186–1,566,355)	3,366.94 (2,873.63–3,880.13)	1,896,622 (1,586,151–2,247,881)	4,184.5 (3,552.91–4,856.13)	2,415,328 (1,898,787–2,963,666)	4,159.12 (3,296.71–5,092.04)	3,044,786 (2,387,913–3,877,694)	3,197.6 (2,143.8–4,581.02)	0.67 (0.64–0.69)
Female	1,112,572 (953,585–1,288,436)	3,333.34 (2,827.51–3,899.6)	1,742,546 (1,526,071–1,957,327)	4,256.1 (3,704.35–4,795.38)	2,363,235 (1,945,354–2,834,300)	4,390.68 (3,607.41–5,251.79)	3,223,297 (2,485,198–4,134,247)	4,012.8 (2,680.9–5,879.07)	1.07 (1.04–1.09)
**Total**	2,414,828		3,639,168		4,778,563		6,268,083		

**Table 4.** Top 5 cancers by prevalence and death rate across provinces (2019).

	Prevalence rate								Death rate							
2019	Pakistan	Punjab	Sindh	Khyber Pakhtunkhwa	Balochistan	Islamabad Capital Territory	Gilgit-Baltistan	Azad Jammu & Kashmir	Pakistan	Punjab	Sindh	Khyber Pakhtunkhwa	Balochistan	Islamabad Capital Territory	Gilgit-Baltistan	Azad Jammu & Kashmir
Breast cancer	1	1	1	1	1	1	1	1	5	5	5	5			5	
Cervical cancer					5											
Colon and rectum cancer			5					5								
Gallbladder and biliary tract cancer														5		5
Kidney cancer									1	1	3	1	1	2	1	1
Larynx cancer				4			5									
Leukemia	4	4		3	3		3									
Lip and oral cavity cancer	2	2	2	2	2	2	2	2								
Multiple myeloma									4	4	2	4	4	3	2	3
Non-melanoma skin cancer													5			
Ovarian cancer	3	3	3	5				3	3	2	4	2	2	4	4	4
Prostate cancer						5										
Testicular cancer						3										
Thyroid cancer	5	5	4		4	4	4	4	2	3	1	3	3	1	3	2

**Table 5.** Leading cancers by incidence, deaths and DALYs across age groups.

Rank	All ages	less than 28 days	28–364 days	1–4 years	5–9 years	10–19 years	20–54 years	55+ years
**Incidence**								
1st	Breast cancer 850,638	Brain and central nervous system cancer 3,160	Other leukemia 11,483	Other leukemia 38,753	Acute lymphoid leukemia 16,183	Hodgkin lymphoma 12,785	Breast cancer 464,182	Breast cancer 377,439
2nd	Lip and oral cavity cancer 545,586	Other leukemia 2,899	Brain and central nervous system cancer 11,453	Brain and central nervous system cancer 24,347	Brain and central nervous system cancer 13,322	Brain and central nervous system cancer 11,393	Lip and oral cavity cancer 258,003	Tracheal, bronchus, and lung cancer 284,910
3rd	Tracheal, bronchus, and lung cancer 385,730	Liver cancer 700	Acute lymphoid leukemia 2,237	Acute lymphoid leukemia 12,212	Hodgkin lymphoma 8,076	Non-Hodgkin lymphoma 9,346	Tracheal, bronchus, and lung cancer 100,009	Lip and oral cavity cancer 276,629
**Death**								
1st	Breast cancer 586,039	Brain and central nervous system cancer 2,843	Brain and central nervous system cancer 3,821	Brain and central nervous system cancer 13,648	Acute lymphoid leukemia 11,774	Acute lymphoid leukemia 8,623	Breast cancer 274,434	Breast cancer 308,256
2nd	Tracheal, bronchus, and lung cancer 392,614	Liver cancer 1,452	Other leukemia 1,319	Other leukemia 7,517	Brain and central nervous system cancer 9,501	Brain and central nervous system cancer 8,151	Lip and oral cavity cancer 145,935	Tracheal, bronchus, and lung cancer 299,806
3rd	Lip and oral cavity cancer 358,638	Other leukemia 940	Acute lymphoid leukemia 817	Acute lymphoid leukemia 7,284	Other leukemia 1,569	Hodgkin lymphoma 7,871	Tracheal, bronchus, and lung cancer 92,256	Lip and oral cavity cancer 208,757
**DALYS**								
1st	Breast cancer 15,988,442	Brain and central nervous system cancer 163,431	Brain and central nervous system cancer 250,594	Brain and central nervous system cancer 769,595	Acute lymphoid leukemia 744,134	Acute lymphoid leukemia 484,367	Breast cancer 9,937,700	Breast cancer 5,850,602
2nd	Lip and oral cavity cancer 9,150,040	Other leukemia 56,792	Other leukemia 92,738	Other leukemia 422,587	Brain and central nervous system cancer 605,690	Brain and central nervous system cancer 468,860	Lip and oral cavity cancer 5,187,013	Tracheal, bronchus, and lung cancer 5,181,897
3rd	Tracheal, bronchus, and lung cancer 8,275,555	Liver cancer 54,031	Acute lymphoid leukemia 53,148	Acute lymphoid leukemia 405,924	Hodgkin lymphoma 323,142	Hodgkin lymphoma 441,885	Tracheal, bronchus, and lung cancer 3,061,299	Lip and oral cavity cancer 3,725,601

## References

[1] Sung H, Ferlay J, Siegel RL (2021). Global Cancer Statistics 2020: GLOBOCAN estimates of incidence and mortality worldwide for 36 cancers in 185 countries [Internet]. CA A Cancer J For Clinicians.

[2] Vineis P, Wild CP (2014). Global cancer patterns: causes and prevention. Lancet.

[3] Bray F, Laversanne M, Sung H (2024). Global cancer statistics 2022: GLOBOCAN estimates of incidence and mortality worldwide for 36 cancers in 185 countries. CA Cancer J Clin.

[4] Cao W, Qin K, Li F (2024). Comparative study of cancer profiles between 2020 and 2022 using global cancer statistics (GLOBOCAN). J Natl Cancer Cent.

[5] Dee EC, Wu JF, Feliciano EJG (2025). National cancer system characteristics and global pan-cancer outcomes. JAMA Oncol.

[6] Hassan M, Butt ZA (2022). Cancer research in Pakistan: opportunities, challenges and the way forward. J Cancer Policy.

[7] ScienceDirect (2024). Pakistan’s Health System: Performance and Prospects After the 18th Constitutional Amendment.

[8] Tufail M, Wu C (2023). Exploring the Burden of Cancer in Pakistan: an Analysis of 2019 Data. J Epidemiol Glob Health.

[9] Anwar N, Pervez S, Chundriger Q (2020). Oral cancer: clinicopathological features and associated risk factors in a high risk population presenting to a major tertiary care center in Pakistan. PLoS One.

[10] Ikram A, Pervez S, Khadim MT (2023). National Cancer Registry of Pakistan: first comprehensive report of cancer statistics 2015–2019. J Coll Physicians Surg--Pak JCPSP.

[11] GBD 2019 (2022). Global, regional, and national burden of colorectal cancer and its risk factors, 1990–2019: a systematic analysis for the Global Burden of Disease Study 2019. Lancet Gastroenterol Hepatol.

[12] The Lancet Global Health (2024). The State of Health in Pakistan and its Provinces and Territories, 1990–2019: a Systematic Analysis for the Global Burden of Disease Study 2019.

[13] Institute for Health Metrics and Evaluation (2024). GBD Results [Internet].

[14] Moore MA, Ariyaratne Y, Badar F (2010). Cancer epidemiology in South Asia-past, present and future. Asian Pac J Cancer Prev.

[15] Cao B, Bray F, Beltrán-Sánchez H (2017). Benchmarking life expectancy and cancer mortality: global comparison with cardiovascular disease 1981–2010. BMJ.

[16] Ding Q, Herrin J, Kryger M (2024). Sex-specific associations between habitual snoring and cancer prevalence: insights from a US Cohort Study. Sleep Adv.

[17] Masood A, Masood K, Hussain M (2018). Thirty years cancer incidence data for Lahore, Pakistan: trends and patterns 1984–2014. Asian Pac J Cancer Prev APJCP.

[18] Xu Y, Gong M, Wang Y (2023). Global trends and forecasts of breast cancer incidence and deaths. Sci Data.

[19] Winn AN, Ekwueme DU, Guy GP (2016). Cost-utility analysis of cancer prevention, treatment, and control: a systematic review. Am J Prev Med.

[20] Wilkinson L, Gathani T (2022). Understanding breast cancer as a global health concern. Br J Radiol.

[21] GBD 2019 Lip O, Collaborators PC (2023). The global, regional, and national burden of adult lip, oral, and pharyngeal cancer in 204 countries and territories: a systematic analysis for the global burden of disease study 2019. JAMA Oncol.

[22] Huang J, Chan SC, Ko S (2023). Disease burden, risk factors, and trends of lip, oral cavity, pharyngeal cancers: a global analysis [Internet]. Cancer Med.

[23] Cogliano VJ, Baan R, Straif K (2025). Preventable Exposures Associated With Human Cancers [Internet]. JNCI J Nat Cancer Inst.

[24] American Association for Cancer Research (2025). Projecting Cancer Incidence and Deaths to 2030: The Unexpected Burden of Thyroid, Liver, and Pancreas Cancers in the United States | Cancer Research.

[25] Bosetti C, Lucenteforte E, Silverman DT (2012). Cigarette smoking and pancreatic cancer: an analysis from the International Pancreatic Cancer Case-Control Consortium (Panc4). Ann Oncol.

[26] Piciucchi M, Capurso G, Valente R (2015). Early onset pancreatic cancer: risk factors, presentation and outcome. Pancreatology.

[27] Momenimovahed Z, Tiznobaik A, Taheri S (2019). Ovarian cancer in the world: epidemiology and risk factors. Int J Womens Health.

[28] Ngwa W, Addai BW, Adewole I (2022). Cancer in sub-Saharan Africa: a lancet oncology commission. Lancet Oncol.

[29] Bray F, Jemal A, Grey N (2012). Global cancer transitions according to the Human Development Index (2008–2030): a population-based study. Lancet Oncol.

[30] Srivastava S, Gray JW, Reid BJ (2008). Translational research working group developmental pathway for biospecimen-based assessment modalities. Clin Cancer Res.

[31] Mudey A, Shukla A, Choudhari SG (2023). Does the graphic health warning on tobacco products have an influence on tobacco consumers in India? a scoping review. Cureus.

[32] Shamsi U, Khan S, Azam I (2020). Patient delay in breast cancer diagnosis in two hospitals in Karachi, Pakistan: preventive and life-saving measures needed. JCO Glob Oncol.

[33] Wu Z, Xia F, Lin R (2024). Global burden of cancer and associated risk factors in 204 countries and territories, 1980–2021: a systematic analysis for the GBD 2021. J Hematol Oncol.

